# Dr. Davey's Mental Pathology
*Contributions to Mental Pathology, with Introductory Observations, containing the past and present State of the Insane in Ceylon, &c., by James George Davey, Licentiate of the Royal College of Physicians, London.


**Published:** 1850-07-01

**Authors:** 


### Art. III.—Dr. DAVEY'S MENTAL PATHOLOGY.*

The general application of the non-restraint system to the treatment of insanity is of so recent date in England, that we were not surprised to learn from the work before us, that the older and less humane method was practised in the distant colony of Ceylon until a few years since.

However, it has pleased the beneficent Ruler of the Universe to

* Contributions to Mental Pathology, with Introductory Observations, containing the past and present State of the Insane in Ceylon, &c., by James George Davey, Licentiate of the Royal College of Physicians, London.

give kind and generous ideas a world-wide currency, so that, in these days, the sweet voice of kindness, speaking all languages, and spreading through all lands, sooner or later obtains a hearing, and teaches all mankind pity and sympathy for the sufferings to which the whole race is subject. Thus it happened that the humane ideas of Pinel and others, accepted by Mr. Elliot, a worthy member of the medical profession, and carried by him to "Lauka's perfumed isle," prompted him, in 1843, to publish in the "Ceylon Observer" some justly indignant remarks on the treatment and condition of lunatics in the Leper Hospital and Jail at Colombo.

The shocking and cruel neglect to which these poor creatures were subjected will be shown by the following extracts from a Report published at the time:—

"In the centre of the brick floor of each cell there is a small grating, which *once* opened into a sewer for the receptacle of the filth; but the sewer being now choked, the offence which the eye and nose experience is better conceived than expressed. Approaching a closed door, a keeper called to the inmate, who was immediately heard scrambling up the back of the door to a little barred window over it, out of which he looked, whilst he held himself by the bars. On inquiry, we learnt that he was generally well conducted, but that, having lately struck a woman, he was confined as a *punishment*.

"What language can give any idea of his cell? He certainly had his stone cot and his mat, and needed not, therefore, to lie on the damp floor; but such a floor! covered with the dirt of bats, myriads of which were flying and hanging about the roof; whilst the choked sewer in the centre of the apartment, and the entire want of ventilation, rendered the stench intolerable.

"In another cell almost the same scene was presented, only that the poor old timid man who occupied it appeared to have been forgotten. The filth from his person was allowed to collect, for several days, in the corners of his cell; and the only reason assigned for his being shut up was, that if the lunatic last spoken of were out (which he was not, be it remembered), he would drive this old man into his cell, and shut the door.

"The last case we shall mention is that of a poor old paralytic woman, who could move only her hands, which she feebly raised in a supplicating manner, whilst the tears ran down her shrivelled cheeks. This also is an individual to whom a cot is denied, so that she is obliged to lie on the bricks, without, we believe, being able to turn from one side to the other."

Mr. Elliot's strictures on the preceding cases having roused the attention of the local authorities, the subject was discussed in the Legislative Council in October, 1843, and a Committee of Inquiry appointed. This committee subsequently published a Report, confirmatory of Mr. Elliot's account in every particular.

"In the Leper Hospital," says this Report, "there are nine cells; . . . . and in one, the occupant, Cassim, afflicted with paralysis of the lower limbs, sleeps on the floor, which is damp, and has, in many places, deposits of his own evacuations. The smell in this, as well as in the other cells in which the lunatics are confined, is very offensive. The floor in each slopes towards a grated aperture in the centre of the room, beneath which is a drain, extending along the middle of the cells in one range. Every drain is choked up to the surface of the grating, and in one instance the floor appeared not to have been cleansed, and the offensive deposits removed, for five or six days. The smell of the urine, which can no longer pass off by the sewer, and is absorbed by the bricks, is most offensive.

"The cells are ill-ventilated, and nearly dark when the door is closed. Numbers of bats continually fly about, and their excrement adds considerably to the stench; some of the patients have *their fixed abode* in their cells."

The Report in question concludes as follows:—

"But the point on which your committee would lay most stress, is the appointment of *a person* trained in one of the well-conducted asylums of the United Kingdom, such as Hanwell, who could classify the lunatics; vary the treatment according to circumstances, and not only render the condition of these unfortunate beings a comparatively happy one, but even restore many of them to reason. Perhaps the condition of lunatics in this island is not worse than it was in many of the pauper asylums of the United Kingdom twenty-five years ago; but, looking to the improvements of the system now pursued at home, and to the happy effects resulting from these improvements, your committee feel imperatively called upon to advocate a like amelioration here."

When the matter was thus forcibly brought before it, the Council of Ceylon did all in its power to remedy so disgraceful a state of management: it adopted the Report unanimously, and, acting on the suggestion therein contained, applied at once to Lord Stanley (at that time Secretary of State for the Colonies) for a fit and proper person to act as superintendent of the insane. It seems, however, that the council did not require, nor indeed expect, the appointment of a medical man as superintendent, the colony being already provided with a sufficient medical staff, but wished for an active and intelligent person, well versed in the internal management and domestic economy of a large lunatic asylum—in fact, an experienced clerk or steward.

The council at the same time took into consideration the project of erecting a suitable building for the reception of the insane.

Lord Stanley, on the receipt of the above application, in the spring of 1844, sent an agent to Hanwell Asylum to offer one of the resident medical officers the appointment of superintendent of the insane in Ceylon, with a salary of 500*l.* a-year, board and lodging being provided. Refused by Dr. Begley, it was accepted by Dr. Davey, who left England in July, and arrived in Ceylon in the following December; accompanied by one or two male and two female assistants, whom the Colonial Office had permitted him to take out with him from Hanwell Asylum.

Thus the Council of Ceylon saw, with some astonishment, the arrival of four or five persons instead of one—a medical officer for whom they had not asked, accompanied by a staff for whom there was no employment. "It never rains but it pours," is a proverb and a truth in the East—it was so in this instance: however, the Ceylon government showed itself anything but grateful for the Colonial Secretary's generosity.

We entirely coincide in the propriety of Dr. Davey's appointment —the resident superintendent of a lunatic asylum ought always to be a medical man—and we feel sure that Dr. Davey performed the arduous duties of his office zealously and well; but we question the wisdom of sending out with him a staff of subordinates, unused to the climate, and unacquainted with the language and manners of the natives; for we think that suitable acclimated European attendants, male and female (discharged soldiers and their wives), could readily have been obtained out of our large Indian armies.

Many circumstances conspired to render Dr. Davey's position at Colombo extremely irksome and disagreeable.

He says: "I found that the appointment of a *medical man* was not only not anticipated, but that it was regarded in a very unpopular light, as an infringement of the rights and privileges of both the military and colonial medical services. It was said I was *not* the person wanted nor required by the legislative council; that the secretary of state had done wrong in sending such a person out; that a "*manager*" only was needed, to classify the lunatics, and see to their various domestic wants and accommodation."—(p. 16.) On presenting his credentials to the secretary for the colony, Dr. Davey was directed to report himself to the deputy inspector-general of hospitals, and to conform with the instructions of that officer: his name was omitted in the list of civil servants: the male attendants were useless, the female an encumbrance, left dependant on him for their support, and made a "subject of wit and satire" by the colonists. He was badly quartered and ill kept, and exposed to numberless petty annoyances.

Yet, in spite of all these discouraging circumstances, Dr. Davey maintained his position with great firmness, vigorously contesting every obstacle, and doing all in his power to accomplish the great object of his mission. Notwithstanding the opposition of his enemies, the lukewarmness of his friends, the desertion of his staff, and the supineness of the local authorities, he succeeded in ameliorating the condition of the unfortunate lunatics in Ceylon in a most satisfactory manner. We give the pleasing results of his labours in his own words:—

"I found the insane in Ceylon, in 1844, in neglect and wretchedness, the companions of debtors and criminals, and of the leprous, blind, and maimed.

"I found them in a lazar-house, where filth and corruption, dilapidation and ruin, were alone visible, in some one or more of their protean shapes.

"I found them, I may truly say, in chains and manacles, and made subject to a variety of personal restraints and to solitary confinement—almost perpetual seclusion.

"I leave them (the insane), in 1849, the occupants of a well-built, airy, and commodious building, wherein every available care, attention, and forethought, are employed for their relief and cure.

"I leave them, in 1849, in an establishment set apart for their exclusive reception, where they are not now, as formerly, subject to the presence of the leprous, blind, and maimed.

"I leave them, in 1849, in peace, and in the enjoyment of the various bodily powers which belong to their nature, and free to move hither and thither at their pleasure."

To have gained so good a cause, and to have accomplished so righteous a work, was an ample compensation for all the annoyances to which Dr. Davey had been subjected, and ought, we think, to have soothed and pacified his wounded feelings. But it was far otherwise. His ultimate success seems to have made him only the more bitter against all who had opposed him.

During the present session, a learned member of the House of Commons described a British colony to be a place "where everybody knows everybody, and everybody hates everybody;" and certainly Ceylon seems to be obnoxious to this disagreeable psychological peculiarity. During the entire period of Dr. Davey's stay in the island, from December, 1844, to January, 1849, he was engaged in one continued squabble with Doctors Roe and St. John, the principal civil medical officers. The quarrel originated in Dr. Davey's false and ill-defined position, was continued with pertinacious hostility by the disputants, and carried on in an intemperate manner, highly discreditable to all concerned. As we have heard only one of the parties at issue, we cannot decide who was the most at fault in Ceylon, but we can decide on the propriety of Dr. Davey's publication in England of this detailed history of the dispute. It appears that Drs. Roe and St. John are gentlemen advanced in life, likely, after many years of active service, to leave their bones in that distant land which has been the scene of their labours; who, although their local reputation may be deservedly high, are but little known here at home; and it was, therefore, unseemly and unnecessary for Dr. Davey to print so many pages of elaborate abuse of them.

We are administering strong reproof, but we do it firmly and conscientiously, from a deep sense of the injury inflicted on the dignity and standing of our profession by such a flagrant disregard, on the part of one of its members, of the common courtesies of life.

The absurd pretensions of our medical forefathers, their jealous emulation, their quarrels and vituperative disputes, are immortalized in the satire of Molière; but the "spirit of the age" has happily changed,—suavity, gentleness and forbearance, ought now to mark all the social relations of the members of a profession whose vocation is eminently one of patience and brotherly love.

Having laid before our readers the foregoing condensed account of Dr. Davey's mission, and of the success and misfortunes which attended it,—the relation of which occupies more than half his book,—we will now proceed to notice that part of it to which we suppose the title, "Mental Pathology," is applied.

Dr. Davey is an uncompromising phrenologist, and also a believer in, and practiser of, mesmerism.* The extent to which he carries his belief in phrenology may be estimated by a perusal of the following extracts from his first Report:—

"In this report it is seen that we have employed the words 'mania,' 'melancholia,' 'monomania,' &c. &c.; we have done so, not because we consider their use in the least degree calculated to elucidate the various abnormal phenomena of the mind—but for this reason, the want of a correct and classical nomenclature of mental diseases. In no department of pathology do we stand in greater need of sound physiological views to direct us than in that one

* See in note (p. 99) the case of a boy cured by mesmerism of epilepsy, but killed soon after by apoplexy.

which treats of cerebral diseases. No person, unless he be a phrenologist, that is, unless he is well acquainted with the functions of the brain in a state of health, can possibly be a good judge of the indications of an unsound mind. To every phrenologist at all accustomed to the insane, the use of such terms as the above must be held to be little else than prejudicial to the advance of science. The location of the various primitive faculties, sentiments, and propensities, by Gall and Spurzheim, and their dependence for a healthy manifestation or otherwise upon distinct portions of cerebral matter, convince us how sadly deficient must our notions of insanity be, when unaided by the facts of this new science (phrenology), or left without the brilliant discoveries of these great men."—pp. 90-1.

"If disease of the brain, like that of the lungs or stomach, or of any other portion of the animal organism, may be, and is more frequently than otherwise, partial—that is, confined to a portion only of the viscus affected, it must follow that the phenomena indicating it must preserve an identity of character, and hence is it, if, for example, the organ of '*self-esteem*' were to take on an abnormal action; its functions would necessarily become impaired in some way. If that portion of the cerebral mass were affected with an exaltation of power, whether dependent on inflammation or not, the fact would become manifest in the increased pride and lofty bearing of the individual so attacked, and conversely; and similarly of the other parts or 'organs' (to write phrenologically) of the brain; of '*acquisitiveness*,' '*destructiveness*,' '*veneration*,' '*benevolence*,' &c. &c. The existence of *moral insanity*, then, as first described, we believe, by Dr. Prichard, is not only confirmed by the light of phrenology, but, what is more, becomes, with its aid, really a matter of demonstration."—p. 92.

"For my own part, I must confess that all my notions of insanity were of the most crude and unsatisfactory nature until I learned phrenology, or the physiology of the brain, as taught by Gall and Spurzheim, and Mr. George Combe; and when I had done so, the abnormal phenomena afforded by my patients at the Hanwell Lunatic Hospital became not only very easily understood, but much more certainly and effectually treated. The mysteries of the mind and of lunacy fade alike into insignificance before the light of phrenological science; with its aid, the brain comes to be regarded as a part and parcel of the human organism, and as subject, in common with the liver and lungs, &c., to similar organic laws and sympathies; the several parts of the *brain*, like the several parts of the *eye* and *ear*, are thus seen to be linked together in one harmonious whole; and just as, in the latter instances, *vision* and *hearing* result from a well-adjusted balance of the several parts of the *eye* and *ear*, and the due performance of their individual and specific functions in either organ, so does *cerebration* (ENGLEDUE) result from or depend upon a well-organized brain, with its several parts duly balanced and adjusted the one to the other. The application of the *principle* herein involved, is sufficient for the complete elucidation of all cases of insanity, of *mental disease,* or my own unaided reflections could never have harmonized so satisfactorily as I have found them to do with the experience of Spurzheim and Combe."—pp. 115-16.

The foregoing passages contain doctrines of some novelty, one of the most positive being, that "no person, unless he be a phrenologist, can possibly be a good judge of the indications of an unsound mind!" Although we are almost afraid to question the correctness of a proposition so boldly stated, we will venture to inquire in what manner phrenology can assist the diagnosis of insanity. Firstly, what is the usual method of "judging of the indications of an unsound mind?" or rather, what are those indications? The first cognizable indication is the external manifestation of such a difference or peculiarity in the language, behaviour, habits, or acts of a person, as show that his mind is not governed by the laws which govern the minds of other men, and which the consentient voice of society decides are the common laws of human nature. The first indication may sometimes coincide with the origin of the disease—the insane state may at once manifest itself in the insane act—as in many cases of impulsive insanity.

But more commonly, the first cognizable indication of insanity is an obvious change or variation in the language, behaviour, habits, or acts of an individual, causing him to differ in conduct, not only from other persons, but also from himself during the former period of his life. Now, whether we judge of a person's insanity, either by simply comparing his state of mind with that of others, or by comparing the *present* state of his mind with its *previous* condition, we are equally compelled to look for some *external* manifestation of the disease. No science can enable us to trace those secret workings of the soul which pass and "give no sign;"—one mind cannot penetrate the mysterious operations of another mind, except by the aid of some exterior evidence. The first process, then, in judging of the indications of an unsound mind, is the perception of those outward visible signs which indicate the unsoundness; the next step, is the comparison of those manifestations with the characteristics of sanity; and upon this comparison the judgment is founded. We cannot clearly see how phrenology alone can aid us here. Phrenology professes to teach that the various primitive faculties, sentiments, and propensities, are located in distinct portions of cerebral matter, and that they are strong or weak in proportion to the greater or less structural development of those portions,—in other words, that there are special organs in the brain for special purposes (as we know there is an optic centre for the sense of sight, and an olfactory for the sense of smell,) and that the functional power of such organs is proportionate to their positive and comparative development.

There are many physiologists, not belonging to the medical profession, who are perfectly acquainted with the healthy structure and functions of the lungs, their positions in the body, their anatomical and vital relations to other parts, who, notwithstanding, are incapable of making an accurate diagnosis of their morbid states; and it seems to us that the phrenologist is in the same position. For phrenology, of itself, can teach merely the relative position and functions of certain parts of the brain—it cannot elucidate their morbid conditions. In the same manner that disease of the lungs is commonly attended by general symptoms—dyspnœa, cough, pain, expectoration, &c.—cognizable to all observers, so is insanity generally indicated by well-marked exterior manifestations, which can be accurately recognised and appreciated without the aid of phrenology. But perhaps Dr. Davey means that, although general indications may suffice to demonstrate the mere existence of insanity, yet phrenology only can enable us to diagnose the precise form and character of the disorder. We suppose that he holds this opinion, not from any explicit exposition of his views, but from his objection to the terms "mania," "melancholia," &c., which he evidently considers to be "words without knowledge." Unfortunately, Dr. Davey, like many other reformers, is satisfied with sweeping away abuses, without providing an efficient substitute in their place; and we have searched through his book in vain for the "correct and classical nomenclature," which he says is so much needed. We, therefore, remain ignorant whether his nosology would be based on the craniological designation of the cerebral organs, or upon the character of the disease affecting them.

Nearly all mental pathologists agree that disorder of the mind may arise from many distinct and opposite conditions of the brain,—from inflammation, from excessive or deficient vascularity, from irritation, either idiopathic or sympathetic, and perhaps from functional impairment. No phrenologist will assert that by its aid we can discover the precise morbid condition of the cerebral substance on which the derangement of its function depends? Can it inform us whether the affected organ is in a state of inflammation, hyperæmia, or anæmia—of idiopathic, sympathetic, or functional irritation? Can it indicate the structure diseased, whether it be fibrous or vesicular nervous matter? Can it determine to which hemisphere of the brain the affected part belongs? To the best of our knowledge and belief it cannot; and Dr. Davey's own observations show the inability of his favourite science to assist us on these points. He says,—

"All those cases, which owe their origin to a *physical* cause, are certainly inflammatory in their nature, and depend mainly on an increased vascularity of a particular portion or portions of the brain, but it is far otherwise with those cases of insanity induced by *moral* causes, so to speak.

"If symptoms of insanity occur in the course of acute febrile diseases or rheumatism, or succeed to a blow on the head, or, in fact, attend on any case either of spontaneous or acquired, that is, of idiopathic or symptomatic inflammation of the brain or its membranes, there is then good reason to infer the diseased cerebration or mental disorder to be the direct effect of such inflammatory condition of the parts within the cranium; whereas if the disease (insanity) succeed to a severe and overpowering moral impression, to any great disappointment or alarm, or to outraged feeling of any kind, involving a sudden, unexpected, and violent shock of the nervous system, through the medium of any portion of cerebral matter, then are we disposed to attribute the phenomena observed, *not* to inflammation, but to *nervous irritation* of the ultimate structure of the brain, including the sensory fibres of the cerebrum,—the afferent and efferent fibres of Foville."—pp. 222, 223.

This, perhaps, is the best method of diagnosis, but it is obviously based on the history and general symptoms of the case; and may be, nay is, serviceably employed, without any knowledge of phrenology.

It has been seen in the last extract, that Dr. Davey considers "*nervous irritation* of the ultimate structure of the brain" the most frequent cause of insanity, but admits that in a certain class of cases it arises from inflammation of the organ. And he seems to regard "exaltation of power"* as a symptom of inflammatory action. This is a doctrine which we cannot allow to pass unquestioned. To assert that inflammation of a cerebral organ—a pathological condition invariably—augments its physiological function, is to contradict the experience of the effects of inflammation on all other organs of the body. We are aware that the incipient stage of inflammatory action,—viz. that of "cerebral determination" followed by a state of "active congestion," is sometimes attended by a seeming exaltation of the functions of the part; but that condition is always temporary, of short duration, and rapidly passes into the destructive stage. When inflammation attacks the eye, it first impairs, and then, if sufficiently violent, destroys vision; so, also, with inflammation of the ear, or of any internal organ. At present, then, it seems to us very im-

* Loc. cit., p. 92.

probable that the effects of inflammation on the cerebral substance should be quite different from its effects on all the other tissues; and until some evidence in support of the doctrine has been adduced, we shall retain the opinion that inflammation always impairs, and, when long continued, destroys the function of the brain.

If inflammation of a particular defined portion of the brain really produced an exaltation of the function of the part—or if, in the language of a distinguished phrenologist, "morbid excitement of the cerebral organs of combativeness and destructiveness may produce raving, violence, and fury, and morbid excitement of the organ of caution produce fear, apprehension, despondency, and melancholy, not from any difference in the *kind* of excitement, but simply from the function of the one being to manifest the propensities first named, and from the function of the other being to manifest the feeling of caution" (*Combe*), it is obvious that phrenology would be of the highest value in the diagnosis of insanity, and we can only regret that so satisfactory a theory should rest on no better foundation.

The objection we have raised to Dr. Davey's views of the effects of *inflammation* on the brain, apply equally well to his views on *irritation* of that organ. For Dr. Davey regards "exaltation of power," or increased functional activity, as the special symptom common to both affections, distinguishing the one from the other by reference to the history and general symptoms of the case. Concerning the effects of irritation on the structure of the brain, our author makes the following judicious remark:—

> "If such an abnormal state of the cerebral mass remain unrelieved, nothing is more likely than the occurrence of inflammation of the brain and its membranes: a state of things which takes place generally in the progress of lunacy in the most stealthy and insidious manner, and which, therefore, continuing unchecked, almost necessarily induces those *palpable* disorganizations of structure, effusions, &c., we have above noticed. Such, we repeat, are, more frequently than otherwise, the effects of *insanity*, and NOT its first *cause*."—pp. 223, 224.

Here Dr. Davey is consistent and logical; for if it be true that irritation of a cerebral organ augments its functional activity, it ought, according to Gall, also to increase its structural development. The sequence of the morbid phenomena would be as follows:—irritation of a cerebral organ exalts its functional power, which in its turn excites its structural development; the augmentation of structure demands an augmented nutrition, hence an increased vascularity, which, in an organ already disordered, readily passes into inflammation. It is therefore probable that irritation of the brain cannot long obtain without congestion or inflammation supervening; and we believe that congestion or inflammation of the cerebral organ cannot long continue without causing an impairment or destruction of its functions.

Returning to the question of the practical utility of phrenology in the diagnosis of insanity, we cannot altogether confirm Dr. Davey's commendation. We think we have shown that by its unaided efforts we cannot correctly judge of the indications of an unsound mind, and that it cannot afford the least assistance in ascertaining the *precise nature* of the morbid condition from which the unsoundness results. And as the efficacy of treatment immediately depends on the precision of diagnosis, it follows that an exclusively phrenological view cannot help us much in the treatment of insanity. The moral treatment must be mainly conducted on psychological principles deduced from experience—the physical must vary in accordance with the patient's general condition, and the presumed state of the diseased organ. We conclude this part of our notice in the words of Esquirol, aptly quoted by Dr. Davey: "What then shall we think of the rash pretensions of those who assume that they can *fix* upon the diseased portion of brain, judging merely from the *character* of the disease?" —(p. 228.) But we have not yet quite done with Dr. Davey's phrenology. By its aid, he thinks to have reduced psychology to the condition of a simple science. He says, "*the mysteries of mind and of lunacy fade alike into insignificance before the light of phrenological science!*" This enthusiastic language is calculated to make all psychologists sigh for a few stray beams of the wondrous lamp or illuminator. Dr. Davey himself has long luxuriated in the full effulgence of its rays, and yet, strange to say, the perusal of his book has not made "the mysteries of mind and of lunacy" any clearer to us than they were before. However, that may be owing to our dulness, want of capacity, or faith; or, possibly, the light is not transmissible. In our darkness, we should have been thankful for a few plain, well-reported cases, exemplifying the use and value of the phrenological psychoscope in the diagnosis and treatment of insanity. After so great a cry, we have a right to demand a sample of the wares.

We will now turn to our author's observations on the jurisprudence of insanity. Here, at the outset, we are startled by the following declaration:—

"*Lunacy* may, and does, generally exist without any impairment of the *intellectual faculties*, and therefore the bare idea even of measuring the responsibility of an individual, reputed of unsound mind, by his understanding, or, in other words, by his capacity to distinguish between right and wrong, is both philosophically and morally incorrect."

If, by the foregoing, Dr. Davey means to proclaim that the majority of insane persons—say 65 in 100—exhibit no impairment of intellect, we beg to state most distinctly that our experience differs from his. We do not say that insanity impairs the intellect immediately in every case; but, according to our observations, the disorder rarely, if ever, exists for any length of time without the intellectual faculties being disordered in a greater or less degree. Such, too, is the opinion of Dr. Conolly, who says:—"Insanity *never* exists without such an impairment of one or more of the faculties of the understanding as induces, or is accompanied by, some loss of the power of comparison"—in other words, an insane person does not correctly realize his own position and his relation to persons and things about him—which is undeniably a very serious defect of intellect. Dr. Davey disregards—nay, almost denies, the intellectual origin of insanity. His favourite maxim, two or three times repeated in the course of his work, is this,—"The speech and actions of the lunatic must be regarded only in the light of *symptoms* of the abnormal condition of the *affections* and *propensities;* which, under circumstances of *health* as well as *disease,* impart the *character* to man"—that is, insanity arises from disordered emotions and propensities, the intellectual faculties themselves remaining unaffected. The following extract will more fully explain Dr. Davey's views:—

"The *intellectual capabilities* of a very large number of even the most decidedly insane, and those found most troublesome to manage at Hanwell, are in every way sufficient to the ordinary purposes of life—are in fact, so far as their *understanding* alone is concerned, in *no way altered* from that they originally were. Under circumstances of excitement, of violence, whether or not attended with incoherence or temporary delusive notions, they retain as complete a *consciousness* of everything, and of their speech and actions, as the attendants about them."—p. 121.

It appears from this, that Dr. Davey considers the possession of *consciousness* the proof that the intellect is not impaired. In illustration of his opinion, he refers to the condition of one suffering from hydrophobia, "who, though impelled to the most extraordinary and rabid conduct, still retains a perfect consciousness of all he may do or say." (We did not know before that hydrophobia is a cerebral disease.) Now consciousness is a fundamental principle of the mind, common alike to the intellect, sentiments, and propensities—it is the alpha and the omega of all mental operations, and cannot be wholly lost except by the total destruction of the entire mind. The imagination is unable to form an idea of a living being permanently deprived of consciousness. "If we are in any way sentient," says Mr. Mill—"that is, have any of the feelings whatsoever of a living creature, the word 'conscious' is applicable to the feeler, and consciousness to the feeling." We may rest assured that consciousness exists so long as the least indication of mind is present. The most miserable idiot is conscious—the new-born infant is conscious. Consciousness may be temporarily suspended, but it returns with the first manifestation of restored animation after syncope, or a fit of apoplexy, epilepsy, or hysteria. We are conscious of the physical properties of matter—of the truth of a mathematical demonstration—of the harmony of music—of the proportions of a statue—of the beauty of a landscape—of the scent of a flower. We are conscious of joy or pleasure at certain events, of grief or vexation at other events. We are conscious of antipathy against some persons, of affection for others. We are conscious of pain, of hunger, of thirst, of fatigue, of the want of sleep, and of the sexual impulse. It is, therefore, evident that consciousness is not exclusively an intellectual principle.

But perhaps Dr. Davey does not employ the word consciousness in its strict psychological signification; and when he says that insane persons are always conscious, means that "they know what they are about." In this sense we partly agree with him; for we are convinced that many of the insane are perfectly conscious of all they do. Whatever may be the motives of their acts, they are fully sensible of the nature, tendency, and probable effect of those acts. When Daniel M'Naughten fired a pistol at Mr. Drummond, he knew the nature of the act he was committing: he was conscious of the "intent to kill," and was aware of its legal consequences; but that does not prove that his intellect was unimpaired, nor invalidate the positive evidence that he was labouring under a delusion, which he had not sufficient intellectual power to correct. Dr. Davey makes the intellect the slave "of the affections and propensities" (p. 125); we, on the contrary, consider it their master: were it not so, how trifling would be man's psychal superiority over other animals. To deny the controlling power of the intellect over human actions would be to renounce the special privilege of our race. Moreover, Dr. Davey's separation of the operations of the emotions from those of the intellect, and his theory of their independent actions, is speculative and artificial, and contrary to true psychology. In the words of that distinguished physiologist, Dr. Todd—

"If a certain part of the brain be associated with emotion, it is plain that that part must be in intimate connexion with the seat of change in the operations of the intellect, in order that each may affect the other; that the former may prompt the latter, or the latter excite or hold in check the former."*

Respecting the question of the moral or legal responsibility of a lunatic, we have to remark that the law virtually acts upon the assumption, that the intellect is impaired in every case of insanity. The fifteen judges decided, in 1843, that "before a plea of insanity should be allowed, undoubted evidence ought to be adduced that the accused was of *diseased mind*, and that at the time he committed the act he was not conscious of right or wrong"—thus requiring proof of two conditions: 1stly, that the accused was insane; and 2ndly, that he was unconscious of right or wrong. And although the *theory* of the law unquestionably makes the latter the turning point of the verdict, still the common *practice* of the law rests the decision on the former point, and is ever willing to take the second for granted, when the first is clearly proved. Indeed we cannot call to mind a single instance in which the court has proceeded to try the responsibility of an accused person, after sufficient evidence had been adduced to show that he was of diseased mind. For some years past there has been a growing inclination among lawyers to evade the question of an alleged lunatic's responsibility, and to confine the issue to the simple question of his sanity; and we confidently anticipate that this will soon become the sole recognised practice. In the meantime, let us not condemn our laws as inhuman, nor attack a procedure of criminal justice more impartial and more favourable to the accused than any other yet constructed and perfected by the wisdom and ingenuity of man. And let Dr. Davey beware of injuring the cause of humanity whilst thinking to serve it, for such seems to us to be the tendency of his opinions. So that, even supposing *it true* "that lunacy may, and does generally, exist without *any* impairment of the intellectual faculties" (p. 91), we should be afraid to develop the doctrine on account of its inevitable result. To teach a jury that insanity does not destroy man's capacity to distinguish between right and wrong, is to take away the special plea for the criminal lunatic's acquittal: for it is upon this principle—the consciousness of right and wrong—that every scheme of retributive justice, both Divine and human, is based.

* Todd and Bowman's Physiology. Vol. i. p. 356.

Not satisfied with promulgating his own views, Dr. Davey falls foul of those who have had the misfortune to differ from him in less enlightened days. He says, "The bar and the bench alike continue to *grovel in the mire and obscurity* which characterized the public acts of *such men* as Mansfield, Erskine, Denman, Abinger, and Follett"—a superfluity of abuse, both undignified and uncalled-for, and peculiarly misapplied to Lord Erskine, whose great sagacity enabled him, even without the aid of phrenology, to point out the true criterion of the reponsibility of the insane—namely, the insanity itself. We also notice that Dr. Davey speaks flippantly of "*one* Lord Hale," an affectation, applied to so great a jurist, philosopher, and citizen—who, in times of violence and anarchy, most nobly represented

"The bodied majesty of England's law;"

and who is not the least worthy in that long roll of learned, upright, and virtuous judges, which is justly regarded as one of the chief glories of our country.

Passing over our author's assault on the originality of Dr. Wigan's theory of the "Duality of the Mind," we turn to the following passage, which shows that Dr. Davey does not confine the peculiarity of his views to one subject alone.

"The *delusions* of the lunatic are always in harmony with the predominant feeling; and do but enable the patient the more clearly to express his *seductive* (?) and abnormal state of mind. I may add, the *delusions* of the insane are *never believed by them,* they are but the morbid colouring to intense and deranged feelings; *a delusion is but a voluntary and tangible ideal of an innate involuntary and morbid impression.*"—p. 95.

How our friends in Westminster Hall would stagger under this definition of the word "delusion."

The author's meaning, if we rightly understand it, is this: the delusions of the insane are voluntary hallucinations, proceeding from involuntary subjective ideas, and consequently are never believed by them. Again we find ourselves at variance with Dr. Davey—firstly, we do not believe that the hallucinations of the insane are voluntary, any more than dreams, or the illusions of delirium; and, secondly, we have learnt, from experience, that the insane have almost always the strongest and most obstinate conviction of the truth and reality of their delusions, during nearly the whole period of their continuance. Every physician, in the least degree acquainted with the insane, is aware of the hopelessness of attempting to eradicate their delusive ideas by any process of reasoning—a proof of the fixity of their belief, and also of the impairment of their intellectual faculties, which renders them incapable of understanding the arguments employed to convince them.

We admit that these delusions most commonly indicate the predominant feeling, in the same way that the note of a musical chord indicates its quality and state of tension; but the delusion cannot be said to be in harmony with the feeling, any more than that the note is in harmony with the chord which produces it. Let us take, for instance, a case of Erotomania, a disordered condition of the faculty of amativeness, in which the affections are bestowed on some real or ideal object: in such a case, the disorder cannot be said to be in harmony with the predominant feeling, being in fact the manifestation of the exaggerated feeling itself. Erotomania is not the harmony of amativeness—the harmonious exercise of that faculty is a rational love for some chosen person of the opposite sex; erotomania is, in the majority of cases, a delusive passion for some ideal or unattainable object—and it is the longing for

"The unreached *phantasy* of their despair,"

which characterizes the mental malady of this class of sufferers.

We think it possible that the belief of lunatics in their delusions may alter, according to inappreciable variations in their state of mind; that the conviction is not so entire at one period as at another; that they may at times even doubt the truth and reality of their delusions; but observation shows, that in such cases it is the doubts which are fugitive—the delusion which is permanent.

Thus, the author of the well-known "*Narrative of the Treatment experienced by a Gentleman during a state of Mental Derangement,*" speaking of his delusions, observes:—"I had a species of doubts, but no one who has not been deranged can understand *how dreadfully true* a lunatic's insane imagination appears to him—*how slight his sane doubts;*" and again, "An insane person is not always aware of anything but his *delusions*, and his delusions contending with his feelings for the mastery over him make him a madman." We conclude with a quotation from the valuable work of the Baron Ernst von Feuchtersleben.*

"It is equally unessential what idea governs the patient, whether it concerns body or mind; whether it be religious, political, or scientific, &c.; the disease consists in this: that some one idea is able to govern him. The idea may be as varied as there are different objects and subjects. It may have reference to the past, the present, or the future; to phantoms of the brain, or to realities; it may be abhorred or cherished. The patient who is seized with fixed

* The Principles of Medical Psychology. Translated for the Sydenham Society.

delusion no longer pays attention to the world beyond his own idea; hence he is glad to flee society in order to indulge, in undisturbed solitude, the congenial, irresistible impulse of his delusion. Every thought and desire of the patient revolve around this fixed delusion, which seizes upon, and, as it were, hurries them along with it."—p. 277.

We have now completed an ungrateful task. It would have been much more agreeable to us to have spoken of Dr. Davey's book in more favourable terms. It came before us with more than an ordinary claim upon our attention. Its author had held an important appointment in this country, and had been preferred to fill an official situation abroad. Consequently, we have given his work our entire attention, and the result is, a firm conviction that it would have been better for Dr. Davey's professional reputation if it had never been published.

---

